# Immune-Mediated Membranoproliferative Glomerulonephritis during Pembrolizumab plus Bevacizumab Therapy in High-Grade Serous Ovarian Cancer: A Case Report

**DOI:** 10.1159/cro/abzag002

**Published:** 2026-08-07

**Authors:** Michel Contreras, Rossana Reina, Jaime González-Montero, Tomás San Martín, Marcela Schultz, Carlos I. Rojas, Mauricio Burotto

**Affiliations:** a Bradford Hill Clinical Research Center, Santiago, Chile; b Finis Terrae University, Santiago, Chile; c Department of Medical Oncology, Clínica Santa María, Santiago, Chile; d Department of Pathology, Clínica Alemana, Santiago, Chile

**Keywords:** Immune checkpoint inhibitors, Membranoproliferative glomerulonephritis, Nephrotic syndrome, Case reports

## Abstract

**Introduction:**

Immune checkpoint inhibitors (ICIs) have transformed oncologic care but are frequently associated with immune-related adverse events (irAEs). When used in combination with anti-angiogenic agents, such as bevacizumab, the risk of renal toxicity may be amplified and requires close monitoring.

**Case Presentation:**

We report the case of a 72-year-old woman with FIGO stage IIIC high-grade serous ovarian carcinoma treated with carboplatin, paclitaxel, bevacizumab, and pembrolizumab. After several cycles, the patient developed grade 2 hypertension, peripheral edema, nephrotic-range proteinuria, and hypoalbuminemia. Renal ultrasound was normal. Laboratory investigations revealed persistent eosinophilia, thrombocytopenia, and nephrotic syndrome. A kidney biopsy indicated immune complex-mediated membranoproliferative glomerulonephritis (MPGN) with severe podocytopathy. Comprehensive immunologic workup excluded differential diagnosis, supporting an ICI-related glomerular disease, potentially exacerbated by bevacizumab. Immunotherapy and anti-vascular endothelial growth factor therapy were discontinued. Renal function stabilized with conservative management, and corticosteroids were not required. Despite an improvement in proteinuria, the disease progressed and the patient was transitioned to a regimen of carboplatin plus pegylated liposomal doxorubicin.

**Conclusion:**

This case highlights the diagnostic complexity and therapeutic dilemma posed by rare renal irAEs, such as MPGN, during combination immunotherapy. Prompt recognition, kidney biopsy, and multidisciplinary management are essential. As the use of combined regimens becomes more common, awareness of glomerular complications is critical to ensure preservation of renal function and guide oncologic decisions.

## Introduction

Immune checkpoint inhibitors (ICIs) have transformed the therapeutic landscape of oncology by enabling durable anti-tumor responses across a wide range of malignancies. These agents – including monoclonal antibodies targeting programmed cell death protein-1 (PD-1), programmed death-ligand 1 (PD-L1), and cytotoxic T lymphocyte-associated protein 4 (CTLA-4) – restore T-cell activity by disrupting inhibitory signaling pathways that promote tumor immune evasion [1]. Their clinical efficacy has been established in multiple tumor types, including ovarian cancers [2]. Despite expanding indications and clinical success, ICIs are associated with a unique spectrum of immune-related adverse events (irAEs), resulting from nonspecific activation of the immune system and loss of peripheral tolerance. These events can affect any organ system, and most frequently involve the skin, gastrointestinal tract, and endocrine glands [3]. Renal irAEs are less common, occurring in approximately 2%–5% of patients receiving ICIs, but they may be underdiagnosed due to nonspecific presentations and delayed onset [4, 5]. The most common renal manifestation is acute tubulointerstitial nephritis (TIN); however, glomerular diseases – including minimal change disease, focal segmental glomerulosclerosis, membranous nephropathy, and membranoproliferative glomerulonephritis (MPGN) – are increasingly recognized [6, 7].

MPGN is a rare but clinically significant manifestation of ICI-induced glomerular injury [8]. Histologically characterized by mesangial proliferation, immune complex deposition, and remodeling of glomerular capillary walls, it often leads to proteinuria and progressive renal dysfunction [9]. In the context of ICIs, its pathogenesis is incompletely understood but may involve T-cell-mediated formation of circulating immune complexes and activation of the classical complement pathway [10]. The risk of renal toxicity is further amplified when ICIs are combined with anti-angiogenic agents such as bevacizumab, a monoclonal antibody that neutralizes vascular endothelial growth factor (VEGF). Bevacizumab is routinely used for ovarian cancer and other solid tumors to impair tumor angiogenesis. However, its inhibition of VEGF signaling can disrupt glomerular endothelial homeostasis, leading to proteinuria, hypertension, and, in rare cases, thrombotic microangiopathy (TMA) [11] Clinical reports suggest that VEGF inhibition may exacerbate ICI-induced glomerular injury by promoting endothelial dysfunction and enhancing local immune activation [2, 12]. Although combination strategies integrating ICIs and VEGF inhibitors have demonstrated promising efficacy in several tumor types, including renal cell carcinoma and gynecologic cancers, the overlapping potential for renal toxicity presents a diagnostic and therapeutic challenge. Early recognition of renal irAEs and histopathologic characterization via kidney biopsy are essential for accurate diagnosis, exclusion of alternative etiologies, and appropriate management.

Here, we report a case of biopsy-confirmed immune complex-mediated MPGN with severe podocytopathy in a patient with advanced ovarian carcinoma treated with ICI and bevacizumab. To our knowledge, this represents one of the few documented cases of MPGN arising in the context of dual ICI and anti-VEGF therapy. This case highlights the importance of vigilant renal monitoring, timely nephrology referral, and individualized treatment decisions in the management of irAEs. This case report was prepared and is reported in accordance with the CARE (CAse REport) guidelines, and the completed CARE checklist is provided as online supplementary material (for all online suppl. material, see https://doi.org/10.1159/cro/abzag002).

## Case Report

We report the case of a 72-year-old Hispanic Caucasian woman, a former light smoker (0.5 pack-years) with a medical history of hypothyroidism and gastroesophageal reflux disease, who presented with progressive abdominal discomfort. A contrast-enhanced CT scan revealed bilateral complex adnexal masses – the largest measuring over 12 cm – accompanied by mild ascites, peritoneal thickening, and supra- and infradiaphragmatic lymphadenopathy. Serum CA-125 was markedly elevated (1,155 UI/L).

Image-guided biopsy of a peritoneal implant confirmed high-grade serous carcinoma of likely gynecologic origin (Fig. 1a–d). Immunohistochemistry showed positivity for CK7, WT1, PAX-8, and aberrant p53 expression. The disease was staged as FIGO IIIC high-grade serous ovarian carcinoma. Following multidisciplinary evaluation, the patient was enrolled in a clinical trial incorporating neoadjuvant chemotherapy and immunotherapy, with planned interval cytoreductive surgery. Over 2 months, she received four cycles of a combination regimen including carboplatin (AUC 6), paclitaxel (175 mg/m^2^), bevacizumab (15 mg/kg), and pembrolizumab (200 mg IV) administered every 3 weeks. She subsequently underwent interval debulking surgery with total abdominal hysterectomy, bilateral salpingo-oophorectomy, and omentectomy. Pathologic assessment confirmed extensive high-grade serous carcinoma involving both ovaries, the right fallopian tube, and omental tissue. Postoperative treatment included five additional cycles of the same systemic therapy, followed by maintenance therapy with pembrolizumab and bevacizumab, with a planned total of 12 cycles.

**Fig. 1.**
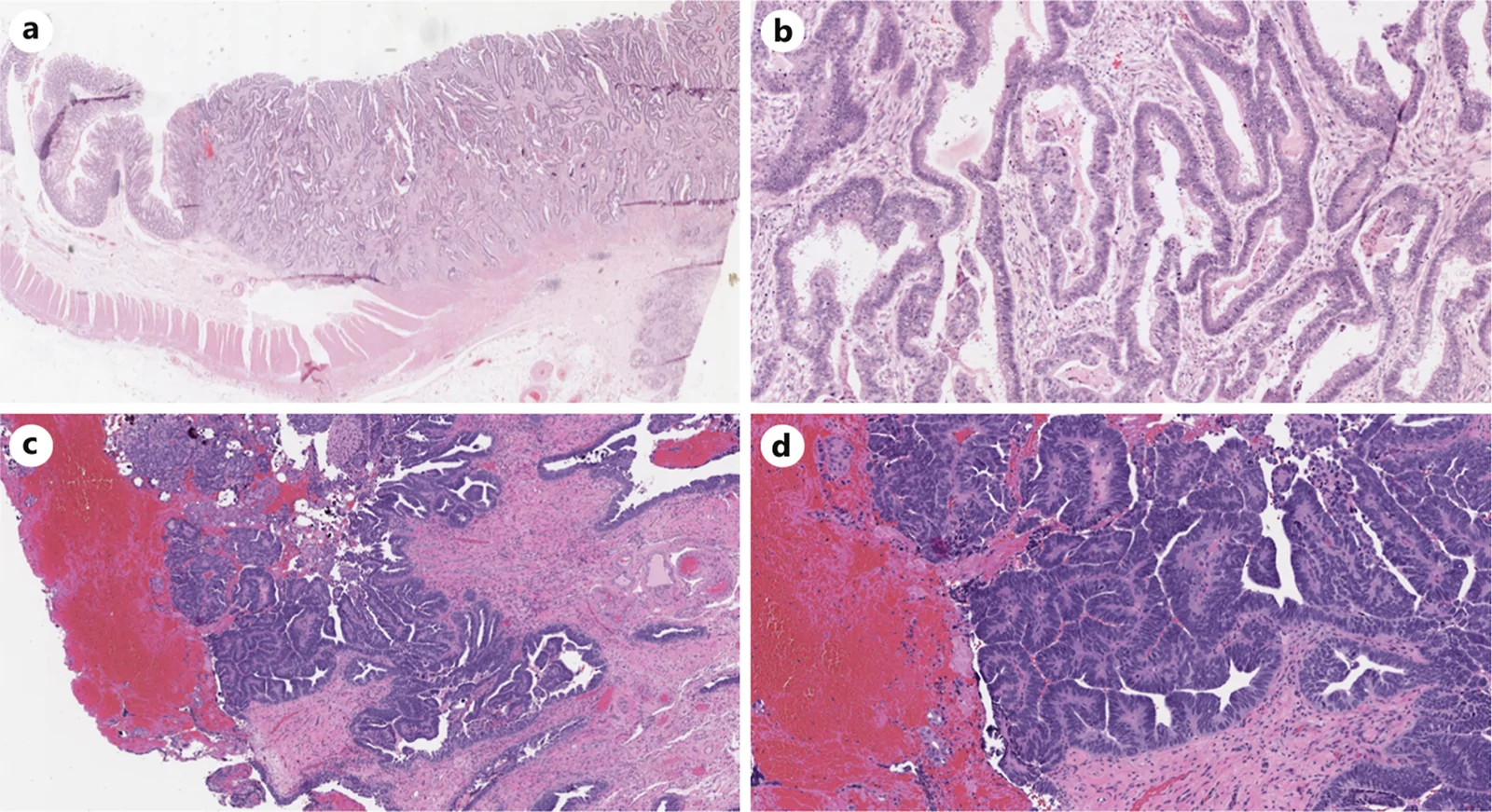
**a–d** Hematoxylin and eosin (H&E)-stained histopathological image showing features of high-grade serous ovarian carcinoma, including marked nuclear atypia, a high mitotic index, and complex papillary architecture.

### Nephrological Complication

During maintenance treatment, approximately 9 months after the initiation of systemic therapy, the patient developed new-onset grade 2 arterial hypertension, bilateral lower extremity edema, and laboratory findings consistent with nephrotic syndrome. These included nephrotic-range proteinuria (5.03 g/24 h), hypoalbuminemia (2.8 g/dL), and dyslipidemia. Immunotherapy and bevacizumab were suspended. Renal ultrasound indicated bilateral nephrolithiasis with preserved parenchymal structure and no evidence of obstruction.

The patient was admitted for further workup. At the time of admission, concomitant medications included levothyroxine, esomeprazole, losartan, amlodipine, alprazolam, and topical 10% urea lotion; none were considered likely contributors to the observed glomerular lesion or suggestive of alternative causes of drug-induced TMA. On examination, she was hemodynamically stable, afebrile, and in good general condition, with moderate pretibial edema. Laboratory studies revealed a serum creatinine of 1.15 mg/dL (baseline 0.9 mg/dL), eGFR 49.2 mL/min/1.73 m^2^, and blood urea nitrogen 18 mg/dL. Additional findings included hyponatremia (129.5 mEq/L), hypercholesterolemia (294 mg/dL), mild macrocytic anemia (hemoglobin 11.5 g/dL, MCV 104 fL), persistent eosinophilia, and thrombocytopenia (platelet count 94,000/mm^3^). Thyroid function tests revealed elevated TSH with low T3. Urinalysis demonstrated 2–5 RBCs/HPF, 0–5 WBCs/HPF, and marked proteinuria (+++), confirmed by quantitative measurement. Supportive management was initiated, including renin-angiotensin-aldosterone system blockade, loop and potassium-sparing diuretics, statins, and anti-hypertensive adjustment. The patient responded favorably, with stabilization of creatinine and resolution of edema.

### Renal Biopsy and Immunological Studies

Given the presence of eosinophilia, thrombocytopenia, and cutaneous rash, TMA was initially considered. A percutaneous renal biopsy was performed alongside an extended immunologic panel. Hemolysis was ruled out, and iron studies were consistent with an inflammatory state.

Light microscopy of the biopsy revealed features of MPGN, including increased mesangial and endocapillary cellularity, obliteration of capillary lumens, intracapillary hyaline thrombi, and dense, non-organized immune complex deposits in the subendothelial and mesangial spaces. Although intracapillary hyaline thrombi were present, overt mesangiolysis, fibrin thrombi, endothelial swelling with marked subendothelial widening, or arteriolar fibrinoid necrosis were not identified. These findings argued against a primary TMA process and favored an immune complex-mediated glomerular injury pattern [11, 12]. Representative low- and high-magnification PAS stains, together with PAM (silver methenamine) staining, are shown in Figure 2a–d. Electron microscopy showed diffuse podocyte foot process effacement in approximately 80% of glomerular capillary loops, consistent with severe podocytopathy (shown in Fig. 3a–c). Immunofluorescence microscopy demonstrated fine granular staining for IgM and both kappa and lambda light chains along the glomerular capillary walls, with intense staining for IgM, C1q, and both light chains within the intracapillary hyaline deposits (shown in Fig. 4a–c). IgG, IgA, and C3 staining were negative. The polyclonal pattern of kappa and lambda light chain staining supported an immune complex-mediated glomerular process and excluded monoclonal immunoglobulin deposition disease. Notably, significant tubulointerstitial inflammatory infiltrates or morphologic features of acute TIN were not identified. The predominant abnormalities were localized to the glomerular compartment, supporting a primarily glomerular pattern of renal injury rather than classic ICI-associated TIN [7, 13]. Autoimmune workup revealed only weakly positive ANA (1:80, granular pattern), with negative anti-dsDNA, ANCA-C, and ANCA-P. IgG was mildly reduced in the absence of hypocomplementemia. Viral serologies for hepatitis B, hepatitis C, and HIV were negative. No monoclonal proteins were identified on serum or urine immunofixation electrophoresis.

**Fig. 2.**
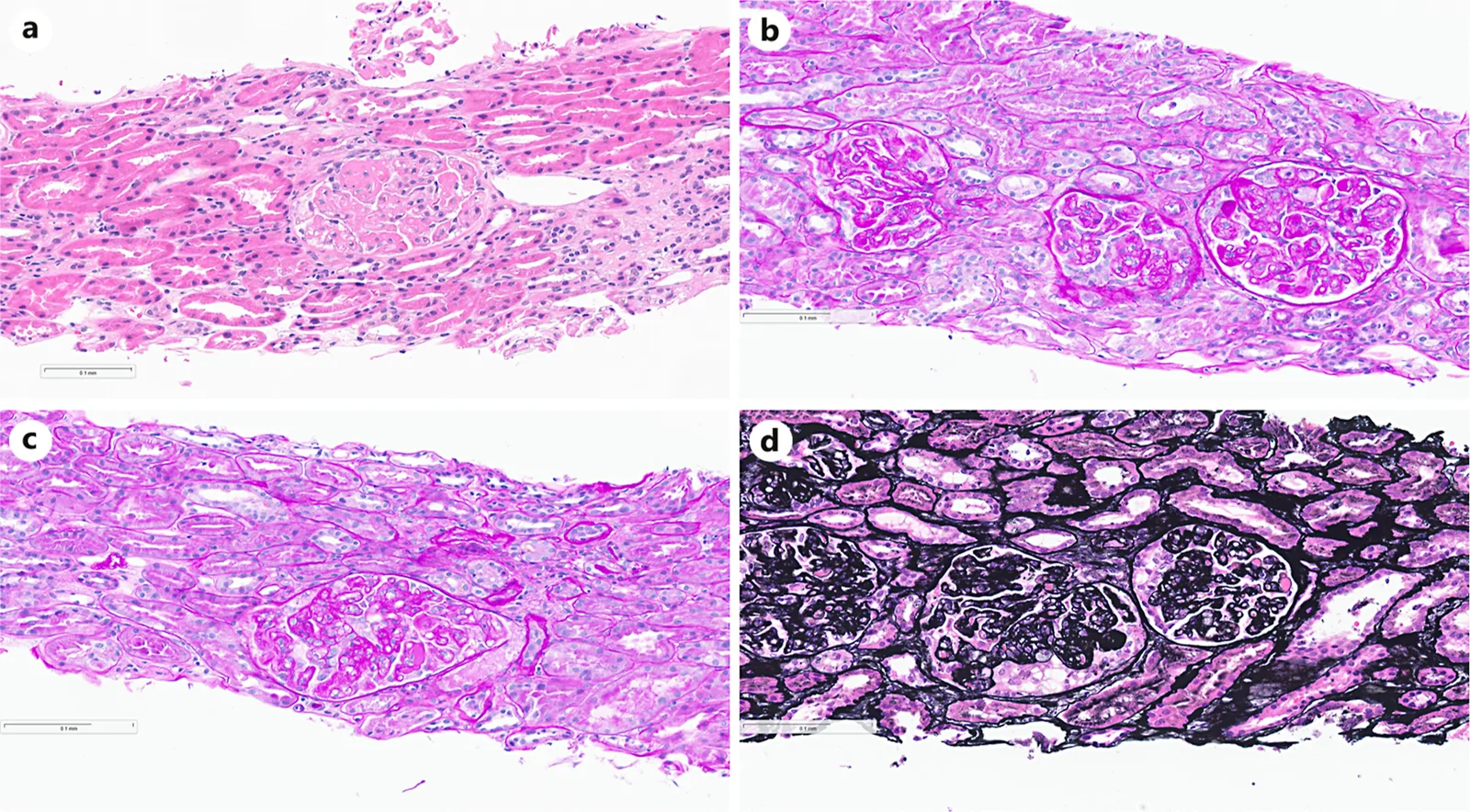
Light microscopy of the kidney biopsy. **a** Hematoxylin and eosin (H&E) stain showing a membranoproliferative pattern of injury with increased mesangial and endocapillary cellularity, partial obliteration of capillary lumina, intracapillary hyaline thrombi, and dense non-organized immune-complex deposits within the subendothelial and mesangial regions. **b** Low-magnification periodic acid-Schiff (PAS) stain demonstrating a membranoproliferative pattern with mesangial and endocapillary hypercellularity. **c** High-magnification periodic acid-Schiff (PAS) stain demonstrating marked mesangial and endocapillary hypercellularity with intracapillary hyaline deposits. **d** Periodic acid-methenamine silver (PAM) stain highlighting glomerular basement membrane remodeling with focal double contour formation, supporting a membranoproliferative pattern of injury.

**Fig. 3.**
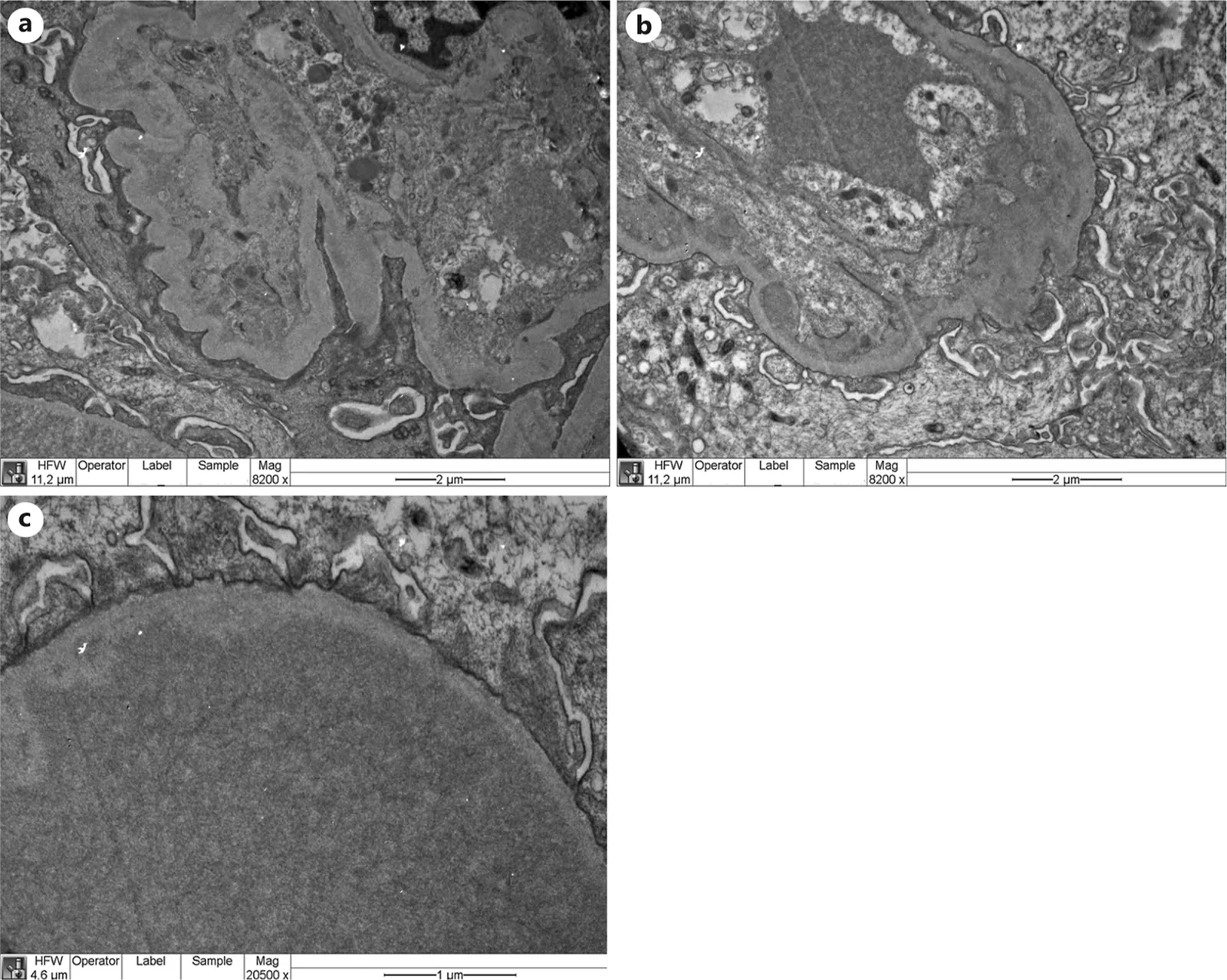
Transmission electron microscopy of the renal biopsy (glutaraldehyde-fixed, epoxy resin-embedded ultrathin sections stained with uranyl acetate and lead citrate). **a–c** A membranoproliferative pattern of glomerulonephritis is shown, with subendothelial and mesangial electron dense immune-complex deposits corresponding to intracapillary hyaline globules and mesangial and endocapillary hypercellularity that obliterate capillary lumina on light microscopy. There is also diffuse podocyte foot process effacement, involving approximately 80% of glomerular capillary loops, consistent with extensive podocytopathy.

**Fig. 4.**
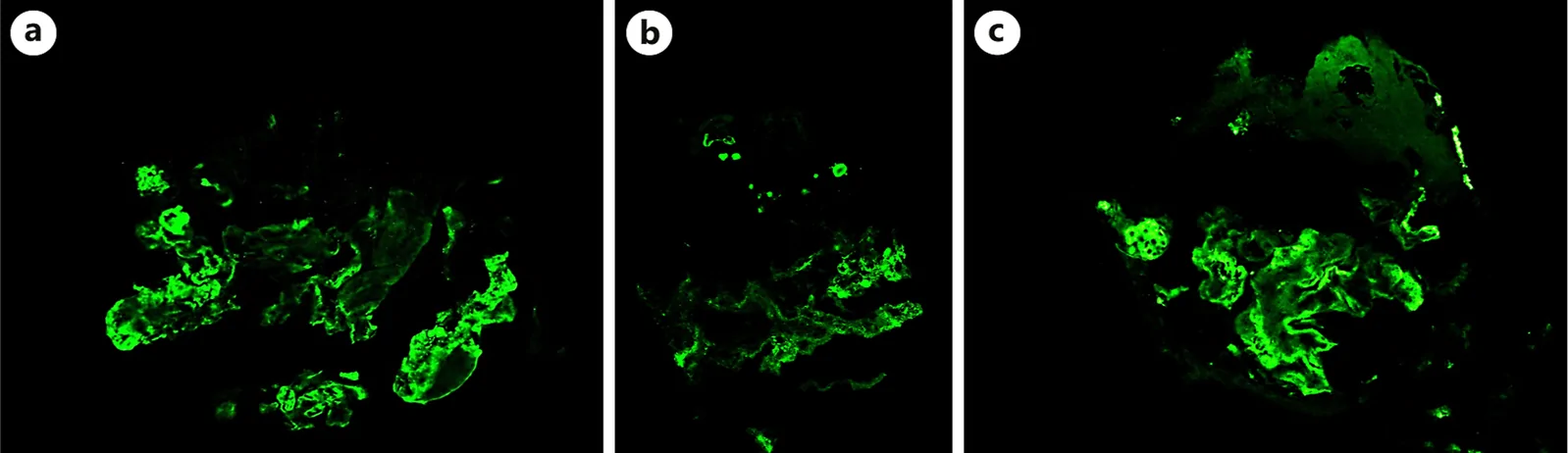
Representative immunofluorescence microscopy of the renal biopsy. IgM (**a**), kappa (**b**), and lambda (**c**) staining demonstrate fine granular positivity along the glomerular capillary walls and intense positivity within the intracapillary hyaline deposits, supporting an immune complex-mediated glomerular process with a polyclonal immunoglobulin deposition pattern.

### Outcome and Follow-Up

Renal function stabilized under conservative management. Final creatinine was 1.45 mg/dL, with an eGFR of 36 mL/min/1.73 m^2^, consistent with renin-angiotensin-aldosterone system inhibition. Proteinuria decreased to 426 mg/24 h, serum albumin rose to 3.3 g/dL, and hyperlipidemia improved with statin therapy. Both eosinophilia and thrombocytopenia progressively improved after drug discontinuation, further supporting the hypothesis of systemic immune activation associated with immune checkpoint blockade. Corticosteroids or additional immunosuppressive agents were not required.

A diagnosis of immune complex-mediated MPGN with severe podocytopathy, possibly reflecting overlapping ICI-associated immune dysregulation and bevacizumab-associated endothelial injury, was supported by the temporal association with treatment exposure, persistent eosinophilia, systemic immune-related manifestations, and exclusion of alternative causes. The concurrent use of bevacizumab was considered a potential exacerbating factor. Both ICI and bevacizumab were permanently discontinued. The patient remained in clinical and radiologic remission for several months following discontinuation of immunotherapy. However, approximately 7 months after diagnosis of the glomerulopathy, imaging studies revealed hepatic, peritoneal, and nodal progression. She was subsequently transitioned to second-line systemic therapy with intravenous carboplatin (AUC 5) and pegylated liposomal doxorubicin (30 mg/m^2^), which was well tolerated.

## Discussion

ICIs have emerged as foundational components of contemporary cancer therapy, significantly improving outcomes for a range of solid tumors, including ovarian cancer [2]. However, the disruption of immune tolerance mechanisms that underpins their efficacy also predisposes patients to irAEs, potentially affecting almost any organ system. Renal irAEs, while less common than dermatologic, endocrine, or gastrointestinal toxicities, can be clinically significant and underrecognized [10]. Among these, glomerular diseases represent a minority of cases but may be particularly challenging to diagnose and manage due to their insidious presentation and potential for long-term sequelae [13]. In the presented case, a patient with FIGO IIIC high-grade serous ovarian carcinoma developed nephrotic syndrome secondary to biopsy-confirmed immune complex-mediated MPGN with severe podocyte foot process effacement, in the context of prolonged treatment with pembrolizumab plus anti-TIGIT and bevacizumab. This presentation is exceedingly rare and highlights a complex interplay between ICI-induced immune dysregulation and anti-VEGF-mediated endothelial injury. The most reported renal irAEs associated with ICIs are acute TIN – accounting for over 90% of biopsy-proven cases [7] – and proteinuria grade 3–5 was reported in only 7 patients in the Keynote 826 trial [2]. In contrast, glomerular pathologies such as minimal change disease, focal segmental glomerulosclerosis, lupus-like nephritis, and MPGN are far less frequently described, but are increasingly reported with the expanded use of ICIs, particularly in combination regimens [6]. A multicenter cohort study reported glomerular lesions in 12% of patients with ICI-related nephrotoxicity, with MPGN being among the rarest histologic patterns observed [4]. Compared with these reports, our case is unusual because it presented as biopsy-confirmed immune complex-mediated MPGN with severe podocytopathy during pembrolizumab plus bevacizumab maintenance therapy, raising the possibility that combined PD-1 blockade and VEGF inhibition may contribute to rare and atypical glomerular irAEs. Although renal irAEs associated with ICIs are increasingly recognized, the true incidence of glomerular toxicities during combined anti-PD-1/PD-L1 and anti-VEGF therapy remains poorly defined, as most available evidence derives from retrospective biopsy-based series and isolated case reports rather than prospective safety analyses. Recent reviews have emphasized that glomerular irAEs represent only a small subset of renal toxicities associated with ICIs, with immune-complex glomerulonephritis and podocytopathies being particularly uncommon [14]. Emerging reports of combined ICI and anti-VEGF therapy have described diverse glomerular patterns, including TMA, mesangial proliferative glomerulonephritis, and complement-mediated injury, suggesting that dual immune and endothelial dysfunction may potentiate atypical renal toxicity [15, 16]. Although current evidence remains limited, emerging clinicopathologic reports suggest that combined PD-1/PD-L1 and anti-VEGF blockade may be associated with more severe or atypical glomerular injury patterns than those classically observed with either therapy alone, potentially reflecting synergistic immune and endothelial dysfunction [12, 15, 16]. The pathogenesis of ICI-associated MPGN is not fully understood but likely involves T-cell activation with subsequent formation of circulating immune complexes and complement activation, leading to subendothelial and mesangial deposition, mesangiocapillary proliferation, and podocyte injury [3].

The contribution of bevacizumab to the renal lesion cannot be overlooked in this case. VEGF plays a pivotal role in maintaining glomerular endothelial cell integrity and podocyte-endothelial crosstalk. Its inhibition by agents like bevacizumab can result in proteinuria, hypertension, and, in rare cases, TMA and collapsing glomerulopathy [11]. Although bevacizumab alone is typically associated with non-inflammatory glomerular damage, there is growing evidence that its combination with ICIs may potentiate immune-mediated injury. This was recently highlighted in a series describing various glomerulopathies – including MPGN – following anti-PD-1 and anti-VEGF co-administration [12]. Nevertheless, several clinicopathologic findings suggested that the renal injury could not be fully explained by bevacizumab-associated endothelial toxicity alone. In addition to nephrotic range proteinuria and intracapillary hyaline thrombi, the patient developed persistent eosinophilia, thrombocytopenia, and cutaneous rash, raising concern for systemic immune activation. Furthermore, the biopsy demonstrated mesangial and endocapillary hypercellularity, dense non-organized subendothelial and mesangial deposits, and extensive podocyte foot process effacement. Comprehensive evaluation excluded alternative causes of immune-complex glomerular disease, including viral infections, systemic autoimmune disease, and monoclonal gammopathy. Although isolated IgM deposition may occur nonspecifically in the setting of subendothelial insudative lesions and was therefore not considered independently diagnostic, the overall clinicopathologic correlation favored overlapping bevacizumab-associated glomerular endothelial injury and possible ICI-associated immune-mediated glomerular disease rather than a purely anti-VEGF-related lesion [11, 12, 14].

Our patient’s presentation with eosinophilia, thrombocytopenia, and nephrotic range proteinuria raised concerns for immune-mediated glomerular disease, which was confirmed histologically. The biopsy showed features consistent with MPGN, including endocapillary hypercellularity, immune complex deposits, intracapillary hyaline thrombi, and widespread podocyte effacement on electron microscopy – findings more consistent with immune dysregulation than with classic anti-VEGF toxicity [12]. Importantly, although intracapillary hyaline thrombi were identified, the absence of mesangiolysis, fibrin thrombi, marked endothelial swelling, or arteriolar fibrinoid necrosis argued against classic bevacizumab-associated TMA and favored an immune complex-mediated glomerular process [11, 12]. Thorough evaluation ruled out primary glomerular disease, autoimmune processes, and infections, supporting a drug-induced etiology.

Interestingly, despite the severity of proteinuria, renal function remained relatively preserved and improved with conservative management alone. Although corticosteroids are commonly employed for ICI-induced nephritis [13], their use in glomerular lesions requires more nuance. In cases with stable renal function, the absence of rapidly progressive features, and a clear response to drug withdrawal, supportive therapy may suffice. This aligns with current recommendations that emphasize tailoring immunosuppressive therapy based on histopathologic findings and clinical severity [13].

The decision to permanently discontinue both therapies was based on the severity of the glomerular lesion and the risk of recurrence. While ICI rechallenge may be considered in selected patients following resolution of mild or moderate irAEs [17], severe, biopsy-proven glomerular disease represents a strong contraindication due to the potential for irreversible damage. Unfortunately, the cessation of immunotherapy was followed by radiologic disease progression, illustrating the therapeutic tension between effective oncologic control and preservation of organ function.

This case contributes to the limited but growing literature on glomerular irAEs in the context of dual ICI and anti-VEGF therapy. It underscores the need for early recognition of renal symptoms, prompt kidney biopsy, and multidisciplinary evaluation. As combination regimens become increasingly prevalent in solid tumor oncology – particularly for ovarian and endometrial cancers – clinicians must remain vigilant for atypical renal toxicities and be prepared to make nuanced, patient-centered treatment decisions.

To better contextualize our findings, we summarized previously reported cases of ICI-associated MPGN or membranoproliferative-pattern glomerular injury in Table 1. Compared with these reports, our case is distinctive because it occurred during maintenance pembrolizumab plus bevacizumab therapy, showed immune complex-mediated MPGN with severe podocytopathy, and improved with drug discontinuation and conservative management alone.

**Table 1.** Previously reported ICI-associated MPGN or membranoproliferative-pattern glomerular injury cases

Author, year	ICI regimen	Cancer type	Renal presentation	Kidney biopsy findings	Management/outcome
Cruz-Whitley et al. [8] (2020)	Nivolumab	Metastatic anal carcinoma	AKI and nephrotic syndrome	MPGN with C3-dominant staining and subendothelial immune deposits	Nivolumab discontinued; renal-directed management reported
Bonilla et al. [18] (2022)	Pembrolizumab	Metastatic lung adenocarcinoma	AKI and significant proteinuria	Immune complex-mediated GN with membranoproliferative and diffuse segmental endocapillary proliferative pattern	Pembrolizumab held; high-dose prednisone, with rapid improvement in kidney function and proteinuria
Yang et al. [19] (2023)	Pembrolizumab	Lung cancer	AKI with urinary abnormalities	C3 glomerulonephritis with membranoproliferative pattern and RBC cast nephropathy	Pembrolizumab discontinued; immunosuppressive/supportive treatment reported
Palamaris et al. [20] (2022)	Atezolizumab	Not specified in renal toxicity series	Renal impairment and subnephrotic proteinuria	MPGN with TMA; IgG, IgA, IgM, and C3 deposition	Immunotherapy withdrawal and immunosuppression were used in the cohort
Present case	Pembrolizumab plus bevacizumab	High-grade serous ovarian cancer	Hypertension, edema, nephrotic-range proteinuria, hypoalbuminemia	Immune complex-mediated MPGN with severe podocytopathy and approximately 80% podocyte foot process effacement	Pembrolizumab and bevacizumab discontinued; renal stabilization with conservative management, without corticosteroids

## Statement of Ethics

This study protocol was reviewed and approved by the Ethics Committee of the Bradford Hill Clinical Research Center, Santiago, Chile, on March 2023. Written informed consent was obtained from the patient for publication of this case report and any accompanying images. The CARE Checklist is provided as online supplementary material to ensure transparent and standardized case reporting.

## Conflict of Interest Statement

The authors have no conflicts of interest to declare.

## Funding Sources

This study was not supported by any sponsor or funder.

## Author Contributions

Michel Contreras: conceptualization, data curation, formal analysis, investigation, visualization, and writing – original draft. Rossana Reina: conceptualization, data curation, formal analysis, investigation, visualization, and writing – review and editing. Jaime González-Montero: conceptualization, data curation, visualization, resources, and writing – review and editing. Tomás San Martín and Marcela Schultz: conceptualization, visualization, resources, and writing – review and editing. Carlos I. Rojas: conceptualization, investigation, data curation, and writing – review and editing. Mauricio Burotto: conceptualization, supervision, data curation, and writing – review and editing. All authors have read and approved the final version of the manuscript and agreed to be accountable for all aspects of the work, ensuring its accuracy and integrity in accordance with the ICMJE criteria for authorship.

## Data Availability

The datasets generated and/or analyzed during the current study are not publicly available due to patient confidentiality, but they are available from the corresponding author upon reasonable request and pending institutional approval.
